# Temporal prevalence and prognostic impact of diabetes mellitus and albuminuria in heart failure with preserved ejection fraction

**DOI:** 10.1186/s12933-025-02708-6

**Published:** 2025-04-05

**Authors:** Nousjka P. A. Vranken, Xinyu Li, Heleen Bouman, Sanne G. J. Mourmans, Anouk Achten, Arantxa Barandiarán Aizpurua, Hans-Peter Brunner-La Rocca, Christian Knackstedt, Vanessa P. M. van Empel, Jerremy Weerts

**Affiliations:** 1 Department of Cardiology, Cardiovascular Research Institute Maastricht (CARIM), Maastricht University Medical Centre (MUMC+), PO Box 5800, 6202 AZ Maastricht, The Netherlands; 2 Division of Nephrology, Department of Internal Medicine, Maastricht University Medical Centre (MUMC+), Maastricht, The Netherlands

**Keywords:** Diabetes mellitus, Albuminuria, Heart failure with preserved ejection fraction, Prognosis, Prevalence, Incidence

## Abstract

**Background:**

Most patients with heart failure with preserved ejection fraction (HFpEF) have a metabolic phenotype in which comorbidities including diabetes mellitus play an important role. Factors related to impaired glucose metabolism, such as kidney disease, may contribute to adverse clinical events. Albuminuria is an early marker of kidney disease. We assessed the prevalence of impaired glucose metabolism and albuminuria in HFpEF over time, and evaluated its prognostic implications.

**Methods:**

Consecutive patients referred to our outpatient clinic and diagnosed with HFpEF between March 2015–November 2023 were included in this study. Patients with type 1 diabetes were excluded. Patients were stratified according to baseline glucose metabolism status (DM + for prediabetes and diabetes, or DM−) and albuminuria status (ALB+ or ALB− for albuminuria > 3.0 mg/mmol and normoalbuminuria, respectively). The primary outcome was a composite of HF hospitalizations (HFH) and all-cause mortality, and was analysed using multivariable-adjusted Cox-regression models.

**Results:**

Among 332 patients with HFpEF (median age 77 years; 67% female), 121 (36.4%) were classified as DM−/ALB−, 106 (31.9%) as DM+ /ALB−, 44 (13.3%) as DM−/ALB+, and 61 (18.4%) as DM+ /ALB+. Both baseline DM and ALB were independently associated with the primary outcome after approximately 3 years: adjusted hazard ratio (aHR) 1.93; 95% confidence interval (CI) 1.25–2.97 and 1.58; 95%CI 1.04–2.41, respectively. Patients in the DM+ /ALB+ group showed the highest risk (aHR 2.85; 95%CI 1.57–5.15). After one year, DM/ALB status was re-evaluated in 250 (75%) patients. New DM+ and ALB+ incidence was 3.9% and 22%in those at risk, respectively. Patients particularly changed ALB groups compared to baseline (n = 63, 25.2%); 27 (10.8%) patients recovered from albuminuria. At 3 years follow-up, the primary outcome mainly occurred in patients who consistently showed albuminuria (27.1%) or who recovered from albuminuria (22.2%), and less so in patients who developed albuminuria after one year (13.9%) or who remained free of albuminuria (8.6%) (*p* = 0.008).

**Conclusions:**

DM and albuminuria are prevalent in HFpEF at baseline, and re-evaluation one year later still reveals new diagnoses. Both factors are independently associated with adverse outcomes. Albuminuria at any time point remains predictive of adverse outcomes in HFpEF.

**Graphical abstract:**

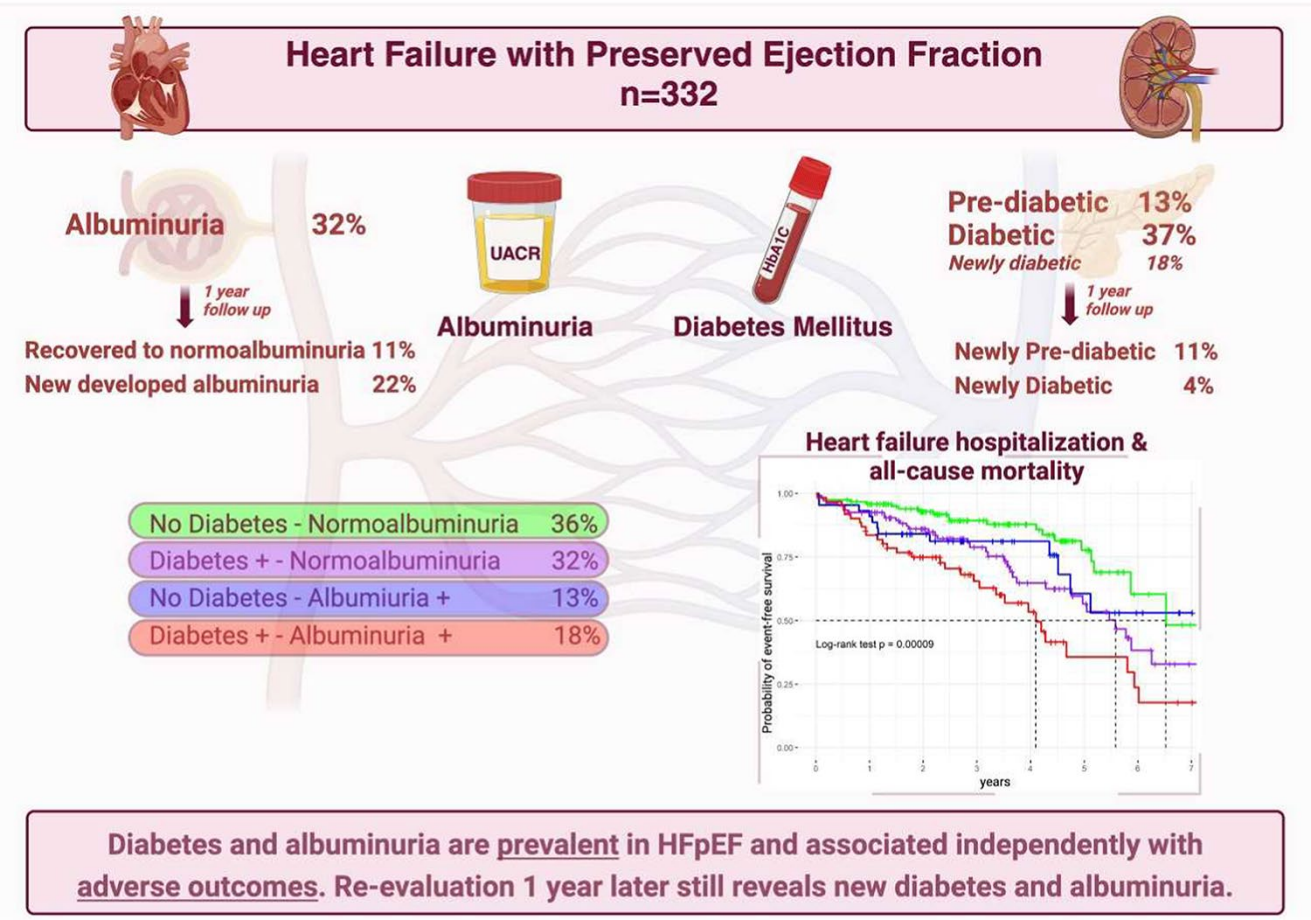

**Research insights:**

**What is currently known about this topic?:**

Diabetes mellitus is an important cardiovascular risk factor in patients with HFpEF, contributing to disease progression and worse outcomes. Albuminuria is a prognostic marker in heart failure patients and more prevalent in patients with diabetes

**What is the key research question?:**

What is prevalence of impaired glucose metabolism and albuminuria in HFpEF over time and how does this translate to prognosis?

**What is new?:**

Both DM and albuminuria each independently associated with worse prognosis in HFpEF. Screening 1 year after HFpEF diagnosis yielded incidence rates of 3.9% and 10.8% for DM and prediabetes, respectively, and 22% for albuminuria. Albuminuria at any time point appeared prognostic in HFpEF, also when albuminuria recovered

**How might this study influence clinical practice?:**

Intermittent screening of HFpEF patients for abnormal glucose metabolism and albuminuria is warranted to optimize risk management

**Supplementary Information:**

The online version contains supplementary material available at 10.1186/s12933-025-02708-6.

## Introduction

Heart failure with preserved ejection fraction (HFpEF) is a cardiovascular syndrome with a rising prevalence, affecting an estimated 30 million people globally [1]. The majority of patients with HFpEF have a metabolic phenotype, characterized by comorbidities such as obesity, diabetes mellitus, and kidney disease [2]. Diabetes has been associated with HFpEF and increased risk of adverse outcomes [3–5]. Impaired glucose metabolism, which encompasses the continuum from normoglycemia to prediabetes to diabetes [6], may drive a significant portion of these prognostic effects, as suggested by the Candesartan in Heart failure Assessment of Reduction in Mortality and morbidity programme (CHARM) [3]. Still, data on the impaired glucose metabolism continuum and its association with adverse outcomes in HFpEF are scarce [7].

Kidney disease, a common consequence of impaired glucose metabolism, may have a bigger impact on adverse outcomes than glucose metabolism status alone [8]. Recent studies have shown that the presence and magnitude of albuminuria are strong prognostic factors associated with HF progression and hospitalizations, irrespective of estimated glomerular filtration rate (eGFR) [9]. Albuminuria is considered an earlier marker of kidney disease than eGFR loss and it is also a marker of microvascular disease (MVD) [10]. As the current working hypothesis puts MVD as a cornerstone in HFpEF pathophysiology [11], albuminuria may be useful to monitor disease progression in HFpEF.

Therapies targeting impaired glucose metabolism and albuminuria include sodium–glucose co-transporter protein 2 inhibitors (SGLT2i), which have showed to reduce HF hospitalizations [12–14]. Moreover, glucagon-like peptide-1 receptor agonists (GLP-1RA) have shown promising results in diabetic and chronic kidney disease patients [15], with future studies awaiting to evaluate its effects in HF patients. A recent study suggests a potential benefit of combining these new therapies to decrease HF hospitalizations in HFpEF patients. [16]

Understanding the interplay between impaired glucose metabolism, albuminuria, and HFpEF is crucial to further optimize employment of these therapies and explore other treatments to improve clinical outcomes. This study assessed the prevalence and incidence of impaired glucose metabolism and albuminuria in a prospective observational HFpEF cohort over time, and investigated their associations with adverse events, to enhance insights into HFpEF metabolic phenotypes.

## Methods

### Study design

A retrospective analysis of a prospective observational cohort study was performed. The study was approved by the Institutional Review Board (NL76585.068.21) and performed according to the principles of the Declaration of Helsinki. All patients provided written informed consent.

### Study population

**Fig. 1:**
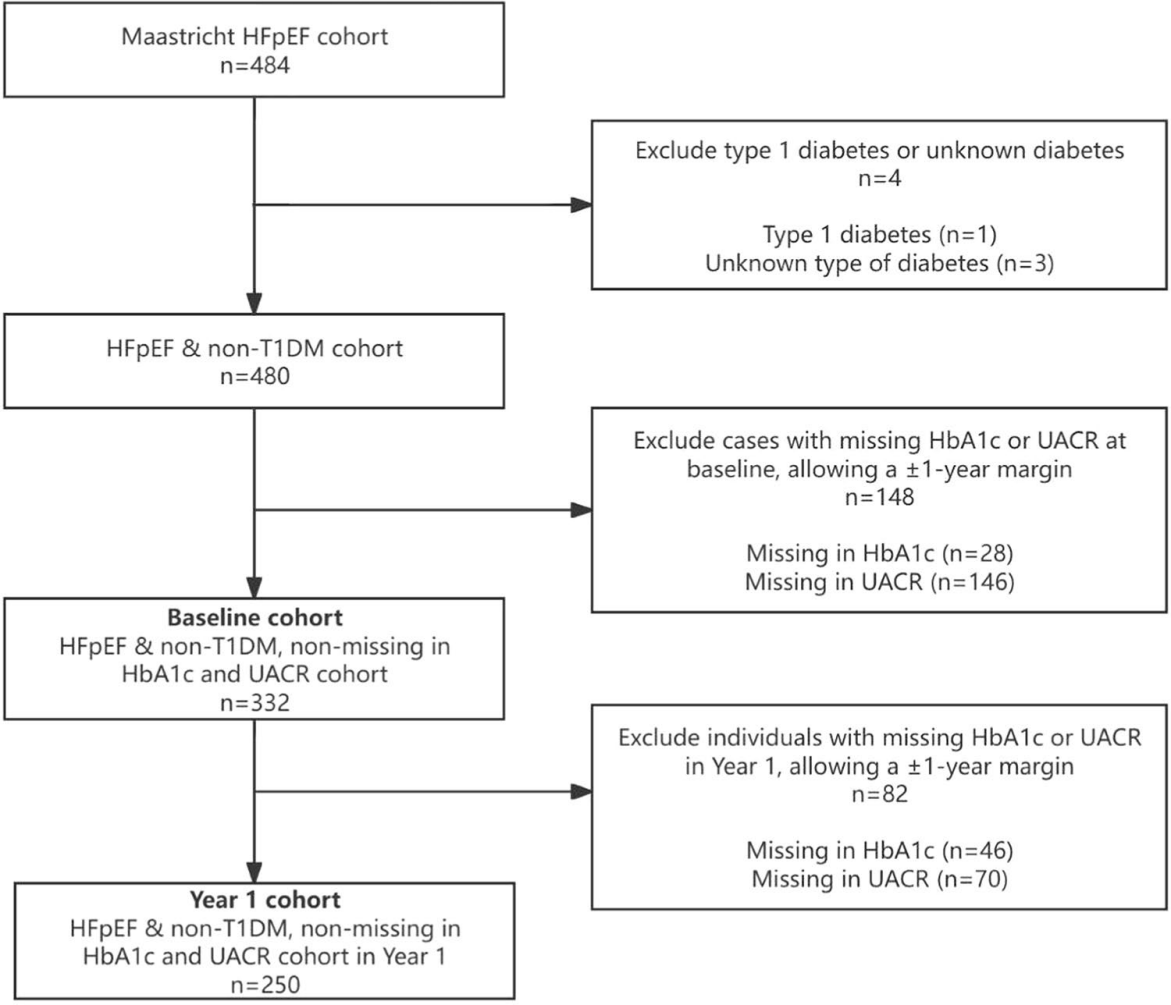
Study flow chart. All patients of the baseline cohort were included for survival analyses until loss-to-follow-up. Measurements were closely timed; for HbA1c, the median was 9 days (interquartile range 8–9 days), and for UACR, the median was 8 days (interquartile range 1–9 days) from baseline visit. Only patients of the 1 year cohort were included in temporal prevalence and incidence of DM/ALB and the accompanied survival analyses. HFpEF: heart failure with preserved ejection fraction, T1DM: type 1 diabetes mellitus, HbA1c: hemoglobin A1c, UACR: urinary albumin-creatinine ratio

Consecutive patients referred to our outpatient HFpEF clinic and diagnosed with HFpEF between March 2015 and November 2023 were prospectively included. All patients systematically underwent a comprehensive diagnostic work-up at baseline including clinical evaluation, echocardiography, biomarker analysis, exercise testing, pulmonary function assessment, and medical history review, as described previously [17, 18]. HFpEF diagnosis was based on the ESC HF guidelines with a consensus of at least two experienced HF specialists [19, 20], and patients received guideline-based pharmacological treatment to optimize their individual cardiovascular health status. Patients were excluded if baseline measurements for hemoglobin A1c (HbA1c) or urinary albumin-to-creatinine ratio (UACR) were missing or assessed later than one year from baseline (Fig. 1). Patients with type 1 diabetes were also excluded.

Patients were stratified by baseline glucose metabolism status (normoglycaemia: HbA1c < 42 mmol/mol; prediabetes: HbA1c 42–47 mmol/mol; and DM: HbA1c > 47 mmol/mol or known diagnosis of DM) and albuminuria status (normoalbuminuria: albumin-creatinine ratio (UACR) < 3.0 mg/mmol; microalbuminuria: UACR 3.0-30 mg/mmol; and macroalbuminuria UACR > 30 mg/mmol) to assess baseline prevalence.

Patients were included for the one year analyses if HbA1c and UACR were present one year later than baseline, with a margin of 12 months.

### Clinical outcomes

Patients were clinically monitored based on routine clinical practice and were also reassessed at the outpatient clinic after one year by research design [18, 20]. Outcomes were evaluated based on electronic health records and patient questionnaires with telephone follow-up consultations, as described previously [18]. The primary outcome was the composite of HF hospitalizations and all-cause mortality. Medication use was recorded at the time of the baseline outpatient visit (and thus already in use at this time), and after one year following the initial visit.

### Statistical analysis

Patients were classified into four categories to overcome anticipated small subgroups: (1) DM+ /ALB+ (diabetes or prediabetes with either microalbuminuria or macroalbuminuria), (2) DM−/ALB+ (normoglycemic with either microalbuminuria or macroalbuminuria), (3) DM+ /ALB− (diabetes or prediabetes with normoalbuminuria), and (4) DM−/ALB−(normoglycemic with normoalbuminuria). At one year follow-up, patients where again classified according to their albuminuria and glycaemic state.

Differences in clinical characteristics between groups were assessed using the chi-square test for categorical variables or analysis of variance (ANOVA) for continuous variables. Results from continuous variables were presented as medians and interquartile ranges, categorical variables by frequencies and percentages. In addition to the baseline analysis, we conducted a one year trend analysis. Sankey plots were used to visualize the one year movement across groups. Patients moving from a DM+ subgroup to a DM− subgroup based on HbA1c levels instead of clinical diabetes diagnosis were still considered DM+ at baseline as well as at one year follow-up.

Kaplan–Meier analysis was employed to assess event-free survival across the four baseline DM/ALB groups, using the log-rank test to evaluate group differences. Cox proportional hazards regression models were used to analyze the independent association of DM and albuminuria status with the composite outcome. The proportional hazards assumption was verified using Schoenfeld residuals for individual variables and the overall model. The models included (1) a crude model, and (2) adjusted for age, female sex, and important clinical prognostic factors in HFpEF: NT-proBNP, eGFR, and atrial fibrillation [21, 22]. Since only eGFR had 4 missing values (1%) and the other covariates had no missing values, a complete-case analysis was conducted, excluding individuals with missing eGFR in the corresponding Cox models.

To explore potential nonlinear relationships, a restricted cubic spline analysis was conducted (hazard ratio of the primary outcome as the dependent variable, either UACR or HbA1c as the independent variable). Four knots were positioned at the 5th, 35th, 65th, and 95th percentiles, as previously described [23, 24]. ANOVA-based tests for nonlinearity were performed using restricted cubic splines. Next, the potential interaction effect between DM and albuminuria was assessed in the final models.

Explorative causal mediation analysis was conducted using the R mediation package. Mediation analysis examines whether a third variable (mediator) explains the relationship between a cause (independent variable) and an effect (dependent variable). To evaluate whether and how albuminuria explains the relationship between DM and primary outcome, we conducted mediation analysis with HbA1c as the independent variable, albuminuria as the mediator, and mortality or HFH as the outcome. This analysis was conducted separately for both baseline albuminuria data and year 1 data. The analysis was performed using logistic regression and an exponential accelerated failure time model, with results computed across 1,000 bootstrapped samples.

Furthermore, event analysis was performed from one year follow-up onwards to evaluate prospective outcomes based on the four DM/ALB subgroups during re-evaluation one year after diagnosis. All statistical analyses were performed using R software (version 4.3.1). A two-sided *p*-value of < 0.05 was considered statistically significant.

## Results

### Baseline

This study included 332 individuals with HFpEF from our outpatient clinic (Fig. 1). Median age was 76 years and two-thirds were female (Table 1). Hypertension was present in most patients and obesity in almost half. Normoglycaemia was found in 165 (49.7%) patients, 44 (13.3%) had prediabetes (HbA1c 42–47 mmol/mol), and 123 (37%) had diabetes at baseline. Microalbuminuria was found in 89 (26.8%) patients and macroalbuminuria in 16 (4.8%) patients. 59 patients did not have a diabetes diagnosis before their baseline visit (35.3% of DM+ patients, 17.8% of the total cohort). Prediabetic patients mostly had normoalbuminuria (n = 32, 72.7%), and less often microalbuminuria (n = 10, 22.7%) or macroalbuminuria (n = 2, 4.5%). Patients with diabetes had a similar distribution in albuminuria, with most patients showing normoalbuminuria (n = 74, 60.2%), followed by microalbuminuria (n = 40, 32.5%) and macroalbuminuria (n = 9, 7.3%).

**Table 1:** Patient characteristics and baseline assessment

	All	DM−/ALB−	DM+ /ALB−	DM−/ALB+	DM+ /ALB+	*p*-value trend
	332	121(36.4)	106(31.9)	44(13.3)	61(18.4)	
Female sex n(%)	221 (66.6)	87 (71.9)	73 (68.9)	28 (63.6)	33 (54.1)	0.101
Age (year)	76.5 [72.1–80.3]	76.8 [72.4– 80.1]	76.5 [71.2– 80.8]	76.2 [72.6– 80.5]	75.9 [72.0– 78.9]	0.757
BMI (kg/m^2^)$, ††,^,*	29.3 [25.9–33.7]	28.1 [24.8– 31.2]	30.4 [27.1– 34.4]	27.3 [25.0– 31.1]	32.0 [28.4–36.7]	< 0.001
*Smoking status*†						0.013
Current smokern(%)	21 (12.3)	5 (8.3)	3 (6.0)	6 (18.8)	7 (24.1)	
Previous smoker n(%)	91 (53.2)	29 (48.3)	32 (64.0)	12 (37.5)	18 (62.1)	
*Glucose metabolism status* $$,††,^^,**						< 0.001
Normoglycaemia n(%)	165 (49.7)	121 (100.0)	0 (0.0)	44 (100.0)	0 (0.0)	
Prediabetes n(%)	44 (13.3)	0 (0.0)	32 (30.2)	0 (0.0)	12 (19.7)	
DM n(%)	123 (37.0)	0 (0.0)	74 (69.8)	0 (0.0)	49 (80.3)	
*Albuminuria status*						< 0.001
Normal UACR n(%)	227 (68.4)	121 (100.0)	106 (100.0)	0 (0.0)	0 (0.0)	
Microalbuminuria n(%)	89 (26.8)	0 (0.0)	0 (0.0)	39 (88.6)	50 (82.0)	
Macroalbuminuria n(%)	16 (4.8)	0 (0.0)	0 (0.0)	5 (11.4)	11 (18.0)	
Hypertension n(%)	250 (75.3)	91 (75.2)	79 (74.5)	37 (84.1)	43 (70.5)	0.454
Significant CAD* n(%)	62 (24.3)	15 (16.0)	17 (21.2)	10 (32.3)	20 (40.0)	0.008
Previous ACS n(%)	39 (11.7)	8 (6.6)	12 (11.3)	7 (15.9)	12 (19.7)	0.057
Previous PCI n(%)	54 (16.3)	16 (13.3)	17 (16.0)	9 (20.5)	12 (9.7)	0.606
Previous CABG n(%)	31 (9.3)	10 (8.3)	6 (5.7)	4 (9.1)	11 (18.0)	0.063
Stroke n(%)	29 (8.7)	11 (9.1)	11 (10.4)	3 (6.8)	4 (6.6)	0.815
AF n(%)	178 (53.6)	58 (47.9)	60 (56.6)	25 (56.8)	35 (57.4)	0.479
Sleep apnea n(%)	65 (19.6)	18 (14.9)	25 (23.6)	4 (9.1)	18 (29.5)	0.022
COPD n(%)	61 (18.4)	21 (17.4)	20 (18.9)	8 (18.2)	12 (19.7)	0.982
*NYHA class* n(%)						0.694
I	9 (2.7)	2 (1.7)	3 (2.8)	3 (6.8)	1 (1.7)	
II	139 (42.0)	56 (46.3)	43 (40.6)	19 (43.2)	21 (35.0)	
III	176 (53.2)	61 (50.4)	57 (53.8)	21 (47.7)	37 (61.7)	
IV	7 (2.1)	2 (1.7)	3 (2.8)	1 (2.3)	1 (1.7)	
*Laboratory assessments*						
Hb (mmol/L)	8.2 [7.5–8.8]	8.4 [7.7–8.8]	8.1 [7.5–8.8]	8.2 [7.6–8.9]	8.0 [7.2–8.6]	0.088
HbA1c (mmol/mol)$$, ††,^^,**	41 [38–49]	38 [35–40]	48 [44–56]	38 [36–40]	49 [44–61]	< 0.001
UACR$,##, ††,^^,%%	1.5 [0.7–3.7]	0.8 [0.5–1.4]	1.1 [0.6–1.6]	6.5 [4.2–10.8]	7.1 [4.0–19.4]	< 0.001
eGFR (mL/min/1.73m^2^)%,†	55 [43–71]	62 [49–73]	53 [41–69]	56 [43–70]	48 [32–70]	0.006
NT-proBNP (pg/mL)	545 [258–1298]	488 [256–1108]	489 [256–1108]	684 [360–1592]	676 [332–1505]	0.080
*Echocardiographic assessment*						
LVEF (%)	60 [57–64]	60 [57–64]	60 [57–64]	61 [58–64]	59 [56–63]	0.544
Peak e’ velocity LV lateral (cm/s)	9 [7–11]	9 [7–10]	9 [7–11]	8 [7–11]	9 [7–11]	0.988
Average E/e’	11 [9–14]	10 [8–13]	11 [9–14]	12 [9–13]	11 [9–15]	0.112
LAVI (mL/m^2^)	45 [37–56]	46 [38–56]	41 [34–57]	48 [40–60]	45 [38–55]	0.118
LVMI (g/m^2^)†,%	78 [64–94]	76 [63–90]	78 [63–88]	84 [71–100]	85 [70–102]	0.003

Medication is detailed separately in Table 3. Bonferroni-corrected significant group differences are depicted between groups using a single symbol for p < 0.05 and a double symbol for p < 0.001;

*/**DM−/ABL+ versus DM+ /ALB+, #/## DM−/ALB+ versus DM−/ALB−, $/$$ DM−/ALB− versus DM+ /ALB−^/^^, DM+ /ALB− versus DM−/ALB+, %/%% DM+ /ALB− versus DM+ /ALB+, †/†† DM−/ALB− versus DM+ /ALB+.

ACS: acute coronary syndrome, AF: atrial fibrillation, BMI: body mass index, CAD: coronary artery disease defined as >70% coronary obstruction, CABG: coronary artery bypass grafting, COPD: chronic obstructive pulmonary disease, DM: diabetes mellitus, eGFR: estimated glomerular filtration rate, HbA1c: hemoglobin A1c, NT-proBNP: N-terminal prohormone brain natriuretic peptide, LAVI: left atrial volume index, LVEF: left ventricle ejection fraction, LVMI: left ventricle mass index, NYHA: New York Heart Association, PCI: percutaneous coronary intervention, UACR: urinary albumin-creatinine ratio.

Subsequently, patients were categorized into four groups, with DM+ concerning both prediabetes and diabetes: DM−/ALB− (n = 121, 36.4%), DM+ /ALB− (n = 106, 31.9%), DM−/ALB+ (n = 44, 13.3%), and DM+ /ALB+ (n = 61, 18.4%). Obesity was more prevalent in DM+ as compared to DM− (*p* < 0.001) (Table 1). Statin and calcium channel blocker (CCB) use differed significantly among the four groups (*p* < 0.05), and were particularly more frequent among DM+ /ALB+ patients. Patients in the DM−/ALB− group showed the highest eGFR and patients in the DM+ /ABL+ group the lowest, with the remaining groups showing intermediate values for eGFR (*p* = 0.006). Patients with ALB+ had higher left ventricular mass on echocardiography compared to ALB−.

### Prognosis

At one year follow-up, 27 (8.1%) patients showed the primary outcome, with 17 (5.1%) patients who had at least one HF hospitalization event. The mortality rate at one year follow-up was 3.6% (n = 12).

**Fig. 2:**
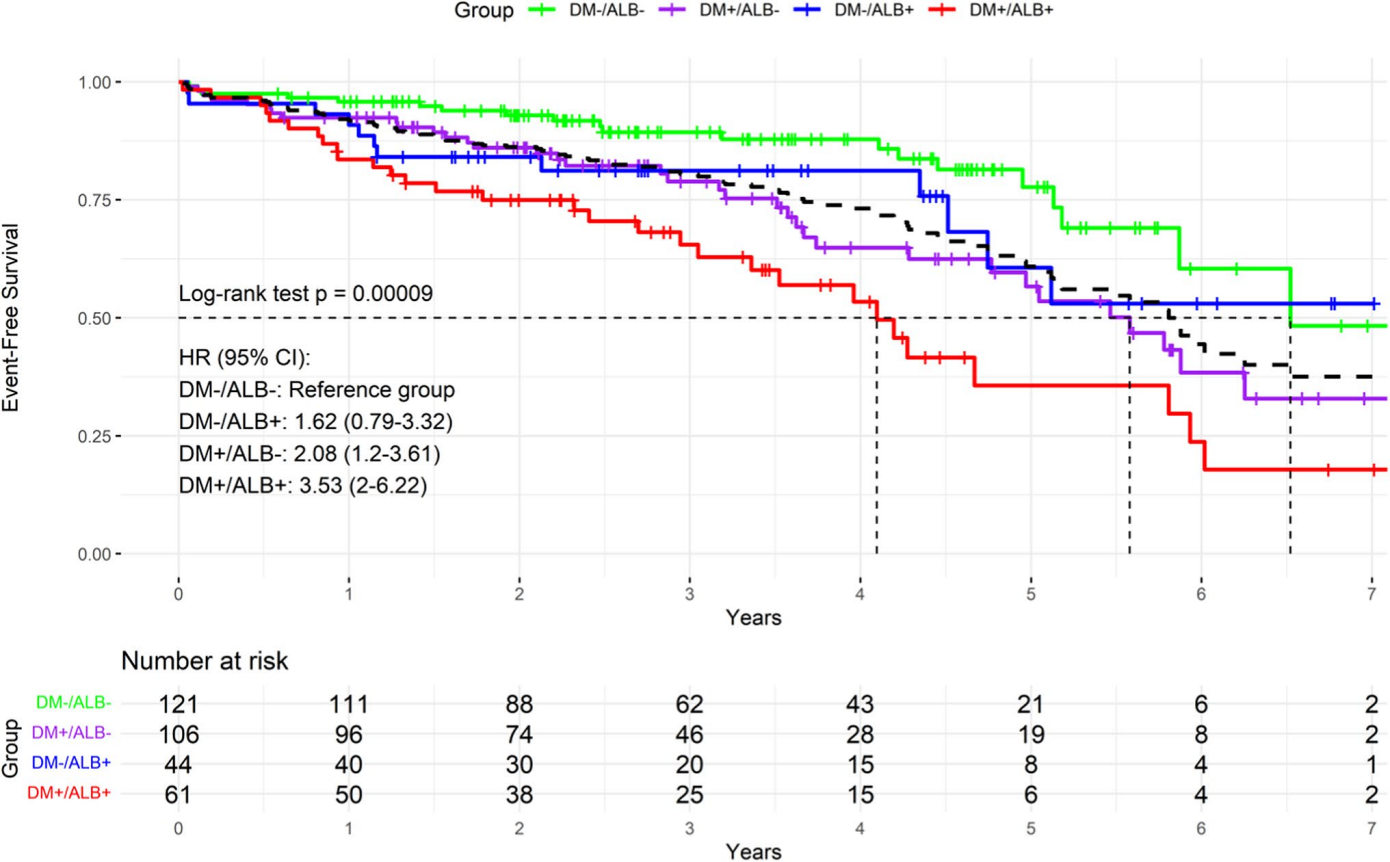
Kaplan–Meier plot for heart failure hospitalization or all-cause mortality in the overall study population and by groups. Sex-specific KM-curves are provided in the supplemental materials. Dotted lines represent 50% of patients with event-free survival. The black dashed line represents the overall study population. The Schoenfeld residuals tests yielded χ^2^ = 1.99 (df = 3, *p* = 0.57), indicating proportional hazards assumption is satisfied globally for the model and assumptions of the log-rank test are met. ALB: albuminuria, DM: prediabetes and diabetes mellitus

After a median follow-up of 2.7 (1.8–4.4) years, the combined outcome of HF hospitalization and all-cause mortality was most prevalent in the DM+ /ALB+ group (n = 18, 29.5%), followed by DM−/ALB+ (n = 8, 18.2%), and DM+ /ALB− (n = 17, 16.0%), and the least prevalent in the DM−/ALB− group (n = 11, 9.1%) (log-rank *p* < 0.001) (Fig. 2). In further analysis, the DM−/ALB− group was used as the reference group and showed the highest survival rates. The DM+ /ALB+ group displayed the worst outcomes (HR 3.53, 95% CI 2.00–6.22). Intermediate survival patterns were observed in the remaining groups.

Diabetes was associated with a consistently increased risk compared to individuals without diabetes across all models, with an HR in the crude model of 2.2 (95% CI 1.44–3.36) and after correction for confounders an adjusted HR (aHR) of 1.93 (95% CI 1.25–2.97), (Table 2). Prediabetes showed to be associated with an increased but non-significant risk, with HR 1.77 (95% CI 0.93–3.39) and aHR 1.47 (95% CI 0.76–2.83).

**Table 2:** Cox regression models of the primary outcome, diabetes and albuminuria combined groups, diabetes, and albuminuria

		Crude model	Fully adjusted model
DM	DM	2.20 (1.44–3.36)	1.93 (1.25–2.97)
	Age (per 1 year)		1.03 (0.99–1.06)
	Female sex		0.56 (0.37–0.85)
	NT-proBNP (per 10 pg/mL)		1.00 (1.00–1.03)
	eGFR (per 100 mL/min/1.73m^2^)		0.89 (0.78–1.00)
	AF		1.19 (0.77–1.84)
Albuminuria	Albuminuria	1.76 (1.18–2.64)	1.58 (1.04–2.41)
	Age		1.03 (0.99–1.07)
	Female		0.58 (0.38–0.88)
	NT-proBNP (per 100 pg/mL)		1.00 (1.00–1.03)
	eGFR (per 10 mL/min/1.73m^2^)		0.88 (0.78–1.00)
	AF		1.28 (0.83–1.97)
By subgroup	DM−/ALB−	reference	reference
	DM+ /ALB−	2.08 (1.20–3.61)	1.93 (1.10–3.36)
	DM−/ALB+	1.62 (0.79–3.32)	1.58 (0.77–3.26)
	DM+ /ALB+	3.53 (2.00–6.22)	2.85 (1.57–5.15)
	Age	1.05 (1.02–1.08)	1.03 (1.00–1.07)
	Female sex	0.58 (0.38–0.86)	0.59 (0.39–0.89)
	NT-proBNP (per 100 pg/mL)	1.02 (1.01–1.03)	1.00 (1.00–1.03)
	eGFR per 10 mL/min/1.73m^2^)	0.82 (0.74–0.92)	0.90 (0.80–1.02)
	AF	1.49 (0.99–2.25)	1.22 (0.79–1.89)

Values are depicted in hazard ratios with 95% confidence intervals. The crude model includes unadjusted univariable analyses. Confounder covariates are included only in the final model to minimize redundancy. The fully adjusted model includes the covariates age, sex, atrial fibrillation, eGFR, and NT-proBNP. The covariates body mass index, significant coronary artery disease, and sleep apnea were not associated with adverse outcomes in either of the presented models.

AF: atrial fibrillation, ALB: albuminuria, DM: diabetes mellitus, eGFR: estimated glomerular filtration rate, NT-proBNP: N-terminal prohormone brain natriuretic peptide.

**Fig. 3:**
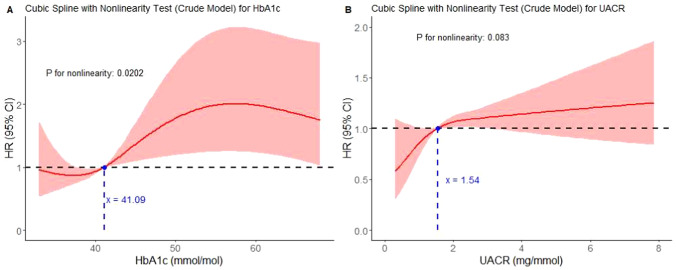
Restricted cubic spline plot of HbA1c and UACR. **A** Crude model for HbA1c, **B** Crude model for UACR. Adjusted results are provided in the supplemental materials (Supplemental Figs. 2 and 3)

The restricted cubic spline plot of HbA1c (Fig. 3A) showed a significant non-linear relationship between HbA1c and the crude HR (*p* for nonlinearity = 0.020), showing a positive HR onwards from approximately 41 mmol/mol, which falls below the prediabetic range of HbA1c range of 42–47 mmol/mol. Hence, the data may be suggestive of prognostic relevance of HbA1c levels in HFpEF from prediabetic range onwards. Albuminuria was associated with a consistently increased risk of the primary outcome across all models (crude HR: 1.76; 95% CI 1.18–2.64, aHR 1.58; 95% CI 1.04–2.41) (Table 2). The restricted cubic spline plot of UACR (Fig. 3B) showed that as UACR increases, the predicted HR rises, with a slight non-linear pattern (*p* = 0.083). A positive HR was found onward from a UACR of approximately 1.5.

No significant interaction was found between DM status and albuminuria for their association with adverse outcomes: interaction term HR 1.04; 95% CI 0.44–2.48, aHR 0.93; 95% CI 0.39–2.25 in the crude model and fully adjusted model, respectively. No significant violations of the proportional hazards assumption were observed for any of the tested models (*p* > 0.05).

### Temporal changes

A total of 250 (75.3%) patients had records available on both HbA1c and UACR at one year follow-up, enabling analysis of DM and ALB subgroups one year from baseline visit. New diabetes was found in 3.9% (based on the population at risk, n = 154), while new prediabetes was found in 10.8% (of 120 patients at risk), and new albuminuria in 22% (of 164 patients at risk).

**Fig. 4:**
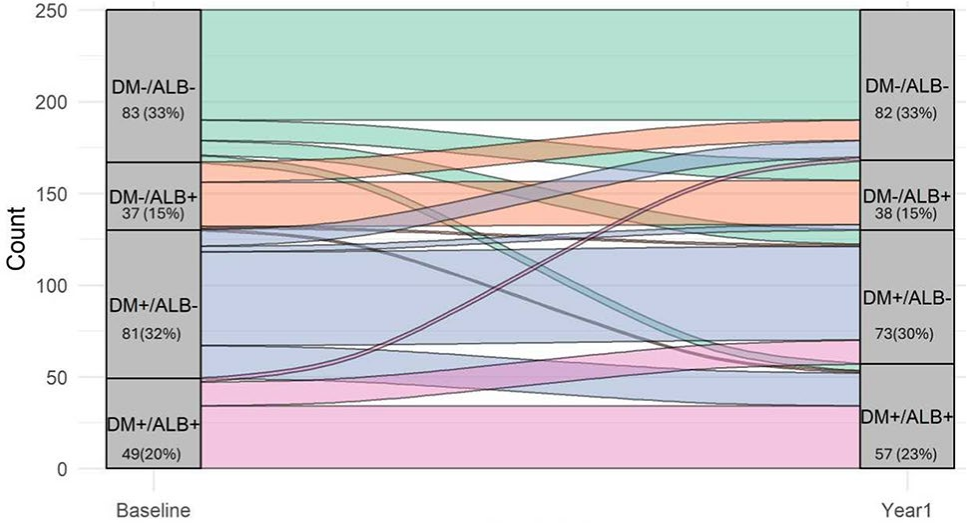
Sankey plot for crossover of groups at one year follow-up. ALB+: albuminuria, ALB−: normoalbuminuria, DM+: diabetes mellitus, DM−: normoglycaemia

A total of 81 patients (32.4%) transitioned to a different DM/ALB subgroup one year after their baseline visit (Fig. 4). Patients particularly changed ALB groups compared to baseline (n = 63, 25.2%).

The majority of patients with DM− at baseline remained in the same subgroup after 1 year, with 14 patients (11.7%, 5.6% of study population at one year) transitioning to the DM+ group, of which most were newly diagnosed prediabetic (n = 13, 92.9%) and one (7.1%) newly diagnosed diabetic (5.2% and 0.4% of study population at one year, respectively). Among 96 patients with DM+ at baseline (either known diagnosis or diagnosed at the baseline visit based on HbA1c), 23 (24%) patients showed HbA1c values corresponding with normoglycaemia, suggesting adequate glycemic control at one year follow-up.

For albuminuria status, most patients with ALB− remained in the same subgroup (n = 128, 78%), while 36 individuals (22% of initial ALB− group, 14.4% of study population at one year) transitioned to the ALB+ subgroup. Among patients with ALB+ at baseline, 27 (31.4%) recovered to ALB− at one year follow-up.

### Temporal changes and prognosis

Within the DM+ group, the prevalence of the primary outcome was similar in those with and without glycemic control at one year follow-up (HbA1c < 42 mmol/L) (n = 97 and n = 21, respectively, log-rank test *p* = 0.820.

**Fig. 5:**
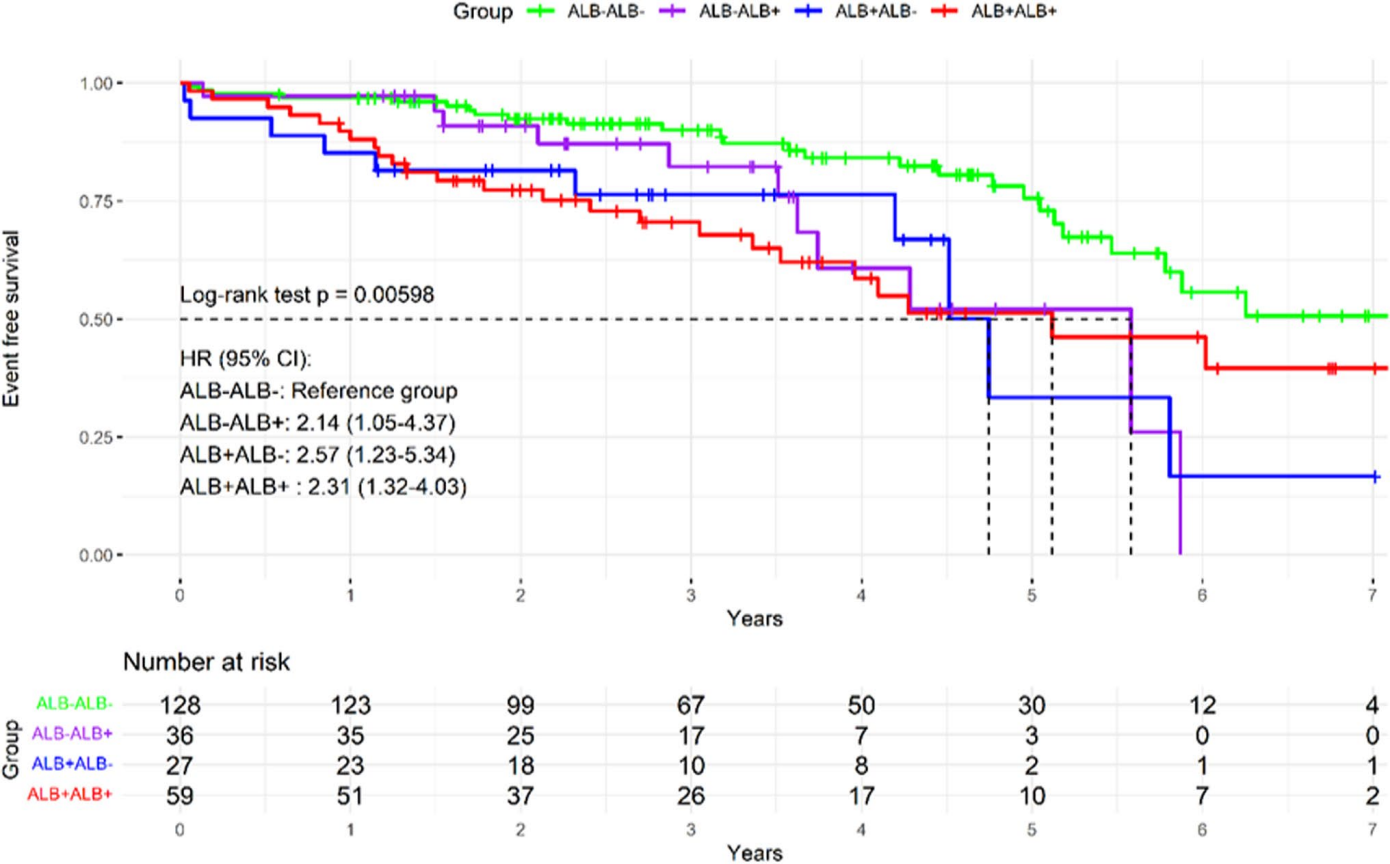
Kaplan–Meier plot for heart failure hospitalization or all-cause mortality in albuminuria transition groups. ALB: albuminuria, DM: prediabetes and diabetes mellitus

At three years follow-up, the primary outcome occurred in 16 (27.1%) of those who consistently showed albuminuria, 6 (22.2%) of patients with recovery of albuminuria, 5 (13.9%) who newly developed albuminuria, and 11 (8.6%) who remained free from albuminuria (Fig. 5, Supplemental Table 1). Comparing outcomes at maximum follow-up between specific subgroups showed no significant difference between those who recovered from albuminuria and those who consistently showed albuminuria (log-rank Bonferroni-corrected *p* = 1.000), or those who developed albuminuria (log-rank Bonferroni-corrected *p* = 1.000). Patients remaining free from albuminuria over time showed the best event-free survival, also compared to those recovering from albuminuria (log-rank Bonferroni-corrected *p* = 0.037). At one year follow-up, NT-proBNP levels were lowest in patients who remained in ALB− throughout time (374 [211–899 pg/mL), were higher in those recovering from albuminuria (743 [299–1480] pg/mL), those who remained in ALB+ over time (771 [410–560] pg/mL), and highest in patients who with developed albuminuria over time (844 [420–1328] pg/mL) *p* < 0.001).

**Fig. 6:**
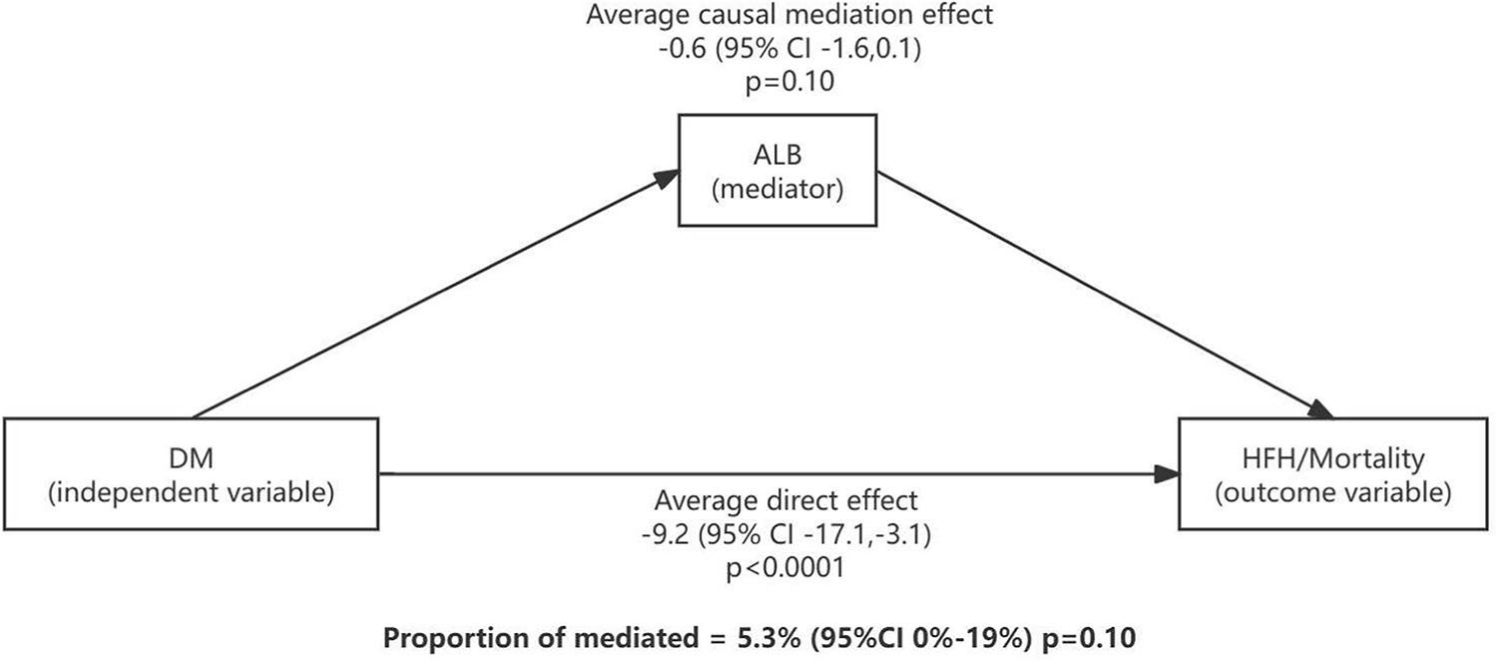
Mediation analysis

Causal mediation analysis indicated a weak mediation effect of albuminuria on DM on the primary outcome (mediation proportion 5.3%, 95% CI 0–19%, *p* = 0.10) (Fig. 6). The mediating effect of albuminuria at one year was further attenuated compared to baseline albuminuria (mediation proportion: 5.5%, 95% CI 0–23%, *p* = 0.16).

### Medication use

Data on medication usage was available in part of the study population. Missing cases were omitted when calculating proportions. Between baseline and one year follow-up, the use of sodium–glucose co-transporter 2 inhibitors (SGLT2i) showed a clear increase over time (0.9% to 12.9%), while angiotensin-converting enzyme inhibitors (ACEi) or angiotensin receptor blockers (ARB) were slightly less often in use (63.6% to 60.5%) (Table 3). In patients who transitioned from ALB+ to ALB− after one year follow-up, ACEi and ARB prescriptions were slightly lower at one year compared to prior the baseline visit (46.7% instead of 59.3%) and SGLT2i were prescribed more often (6.7% instead of 0%, respectively), though the number of available datapoints was limited (Supplemental Table 2).

**Table 3:** Medication use before the first outpatient clinic visit (T0) and after one year follow-up (T1)

		All (n = 332)	DM−/ALB− (n = 121)	DM+ /ALB− (n = 106)	DM-/ALB+ (n = 44)	DM+ /ALB+ (n = 61)	*p*-value
T0	ACEi/ARB	208 (63.6)	74 (62.2)	71 (68.3)	26 (59.1)	37 (61.7)	0.667
	MRA	44 (13.5)	14 (11.8)	13 (12.5)	7 (15.9)	10 (16.7)	0.769
	SGLT2i	3 (0.9)	2 (1.7)	1 (1.0)	0 (0.0)	0 (0.0)	0.631
	BB	232 (70.9)	80 (67.2)	77 (74.0)	29 (65.9)	46 (76.7)	0.428
	Loop diuretic†	192 (58.7)	58 (48.7)	64 (61.5)	26 (59.1)	44 (73.3)	0.015
	CCB†	137 (41.9)	35 (29.4)	47 (45.2)	23 (52.3)	32 (53.3)	0.004
T1	ACEi/ARB	98 (60.5)	37 (59.7)	30 (65.2)	17 (58.6)	14 (56.0)	0.873
	MRA	23 (14.3)	7 (11.5)	8 (17.8)	2 (6.7)	6 (24.0)	0.242
	SGLT2i	21 (12.9)	5 (8.1)	10 (21.7)	3 (10.0)	3 (12.0)	0.192
	BB	107 (66.0)	41 (65.1)	35 (77.8)	13 (43.3)	18 (75.0)	0.014
	Loop diuretic†	106 (65.0)	30 (47.6)	38 (82.6)	20 (66.7)	18 (75.0)	0.001
	CCB†	62 (38.0)	20 (32.3)	14 (30.4)	16 (53.3)	12 (48.0)	0.110

Values are depicted in n (%). Missing values were omitted from the analysis. P-values are determined using the Chi-squared test.

ACEi: angiotensin-converting enzyme inhibitor, ARB: angiotensin receptor blocker, BB: beta blocker, CCB: calcium channel blocker, MRA: mineralcorticoid receptor antagonist, SGLT2i: sodium–glucose co-transporter 2 inhibitor, T0: baseline visit, T1: visit 1 year after baseline.

†Bonferroni-corrected significant group difference *p* < 0.05 for DM−/ALB− versus DM+ /ALB+.

## Discussion

The studied HFpEF cohort included 332 patients from our outpatient clinic. New diabetes mellitus was found in one in six HFpEF patients at baseline. Impaired glucose metabolism (prediabetes and diabetes) and albuminuria were both independent predictors of adverse outcomes, with impaired glucose metabolism as the strongest predictor. Re-evaluation after one year revealed new impaired glucose metabolism and albuminuria in up to one in five patients. Cross-over between subgroups was prevalent during follow-up, mostly from DM−/ALB+ to DM−/ALB−, suggesting an adequate effect of interventions responsible for the resolution of albuminuria. Still, albuminuria at any given timepoint appeared to be associated with more adverse outcomes and higher NT-proBNP levels during follow-up.

Until now, studies combining glucose metabolism status as well as albuminuria in patients with HFpEF are scarce. Selvaraj et al. showed a significant association between the presence of diabetes and changes in UACR after multivariable adjustment [25]. They found that albuminuria was significantly and independently associated with their primary outcome, a composite of cardiovascular death, aborted cardiac arrest, or HF hospitalization. In their multivariable analyses, diabetes was adjusted for and not considered a separate covariate, omitting evaluation of the effects of diabetes besides albuminuria. Katz et al. reported an increasing prevalence of DM alongside rising UACR values, as well as an association between increasing UACR and their composite endpoint of cardiovascular hospitalisation or death [26]. This association was not significant after adjusting for cardiovascular risk factors, including diabetes mellitus, as well as brain natriuretic peptide. In the current study, albuminuria was significantly associated with the primary outcome, in both with and without concomitant diabetes. Yet, diabetes was associated with a higher risk of adverse events, compared albuminuria. In addition, the absence of statistical significance in the interaction term of DM and albuminuria, and no relevant mediator effects on outcome and diabetes from albuminuria at any time point, again underline the independency of diabetes and albuminuria as prognostic determinants in HFpEF.

### Impaired glucose metabolism in HFpEF

The relevance of impaired glucose metabolism for the development and progression of HFpEF may occur prior to clinical DM, and is a noteworthy treatable risk factor. The development of HFpEF due to impaired glucose metabolism may result from altered microvascular function and increased oxidative stress through free radical release, leading to metabolic derangements and reduced myocardial energy efficiency. Both of these factors may contribute to diastolic dysfunction and the metabolic profile characterizing HFpEF [27, 28]. Previous reports found a prevalence of prediabetes in HFpEF around 18–54% and DM around 25–44%, which was associated with increased morbidity and mortality rates [3, 27, 29–31]. The current study aligns with these findings. Moreover, the current results showed an association with adverse outcomes beyond HbA1c levels of 41 mmol/mol—which is even slightly below the accepted HbA1c range for prediabetes. Differences in patient selection might explain the aforementioned range of DM prevalence in HFpEF across studies. More fundamentally, the varying degree of awareness of HFpEF and the subsequent diagnostic challenge may introduce an uncertain degree of selection bias in these studies. The current study cohort included patients from our HFpEF outpatient clinic in which systemic screening of comorbidities takes place routinely, which may have contributed to detection of cardiovascular risk factors such as impaired glycose metabolism in this patient population.

Since around one in six patients with diabetes in the present study did not have their diagnosis before the baseline visit, active screening for impaired glucose metabolism in patients receiving an HFpEF diagnosis is warranted, as is also recommended by the European HF guidelines [20]. At one year follow-up, we still frequently newly diagnosed prediabetes and DM, suggesting that annual screening for DM in HFpEF is important to optimize cardiovascular risk factor management in order to optimize clinical outcomes.

### Albuminuria in HFpEF

Albuminuria likely plays a significant role in disease progression in HFpEF, is easily assessed, and can be treated. The current cohort showed an albuminuria prevalence based on spot urine of one in three, which falls within earlier reported ranges [3, 26]. Albuminuria is known to correlate with renal insufficiency, cardiovascular disease, and an increased mortality risk, both in the general population as well as in diabetic and HF patients [32]. Moreover, the association between albuminuria and end-stage renal disease has been established across all categories of eGFR. In HFpEF patients in particular, albuminuria is thought to be the result of persistent microvascular dysfunction and systemic endothelial inflammation, translating to endothelial dysfunction in the kidneys [34, 35], and associated with left and right ventricular remodeling [26]. The presence of albuminuria might not be attributable solely to the presence of comorbidities including hypertension or diabetes, since the prevalence of microalbuminuria was found to be 6.6% in a general population without these known cardiovascular risk factors [36], while UACR ≥ 30 can be found in 23.9% and 21.7% of patients with diabetes and hypertension, respectively. [37] Venous congestion could also lead to albuminuria in HF due renal venous congestion leading to reduced renal blood flow [38], which might be the reason why patients more easily recovered from albuminuria to normoalbuminuria during follow-up in the present study.

Regardless of the timepoint, albuminuria was associated with more adverse outcomes in the present study. The association between baseline albuminuria and clinical outcomes in HFpEF has been assessed in a limited number of studies [3, 25, 39]. Jackson et al. found a gradual increased risk of mortality and heart failure hospitalizations in increasing values for UACR [39]. The Treatment of Preserved Cardiac Function Heart Failure with an Aldosterone Antagonist (TOPCAT) trial showed similar results. In addition, a 50% reduction in UACR was found to be associated with an almost 10% decrease in the risk of HF hospitalization and all-cause mortality, suggesting an inverse correlation between the extent of albuminuria and the risk of worse clinical outcomes [25]. A recent trial with a non-steroidal MRA in HFpEF, finerenone, demonstrated improved outcomes and reduced albuminuria, but it has not yet been proven that reducing albuminuria was a strong mediator to improve outcomes [40, 41]. The relationship between albuminuria and clinical outcomes using cubic spline analysis in the present study also showed a gradual upward trend and an association with the primary outcome at UACRS levels within the normoalbuminuria range (UACR < 3.0) as defined by Kidney Disease: Improving Global Outcomes (KDIGO) [42], which was also applied in the current study for the classification of albuminuria. These results suggest that, in HFpEF, a clinically relevant extent of albuminuria might be present in the now-considered normal range of albuminuria. Similar findings have been reported, *i.e.*, a high-normal range of UACR (1–2.9; versus UACS < 0.5) already associated with incident HF (aHR 1.91) in the ARIC population study [43]. Moreover, recovery from albuminuria was not associated with improved outcomes in the present study, in contrast to the TOPCAT trial [25], possibly because MRA prescription was infrequent in our cohort. Our data suggest that once albuminuria is present, adequate therapeutic measures are required to reduce adverse outcomes. Future studies should aim to assess clinical outcomes in HFpEF across the spectrum of albuminuria and glucose metabolism and evaluate which effective intervention actually translates to improved clinical outcomes. It is recommendable to take absolute UACR values and glucose metabolism into account, to ultimately define a relevant cut-off value for prognostication and intervention in HFpEF.

### Medical treatment signals

When focusing on medication use throughout the study timeframe, a few trends were observed. Those with macroalbuminuria at baseline were more likely to receive CCB and in case of concomitant DM, loop diuretics, as compared to those with normoalbuminuria. Within the subgroup of patients who recovered from albuminuria to normoalbuminuria in one year, the number of ACEi/ARB and MRA prescriptions remained largely unaffected, and outcomes were similar in those who continued to have albuminuria during follow-up. In the current study, SLGT2i were increasingly prescribed during follow-up, although absolute frequencies were low. As discussed in the previous paragraph, the albuminuria-reducing effect of MRAs may be a mechanism by which patients incur less HF hospitalizations and all-cause mortality [25, 44]. The mechanism by which the use of MRAs leads to a reduction of albuminuria remains incompletely understood. This effect is probably, at least in part, the result of the antihypertensive effects of MRAs, although the reduction in UACR in spironolactone users persisted after adjusting for systolic blood pressure change [25]. Important to note is that the use of either ACEi or ARB at one year follow-up was also associated with a decrease of UACR in the study by Selvaraj et al [25]. This could have contributed to the presented outcomes, since ACEi and ARBs, collectively referred to as renin–angiotensin–aldosterone system inhibitors, confer to the most commonly applied therapy for albuminuria. Unfortunately, their study was underpowered to conduct a mediation analysis.

In addition to ACEi, ARB, thiazide diuretics [45], and more recently embedded in European guidelines, SGLT2i, are part of pharmacologic treatments to reduce albuminuria. The latter have become an integral part of HF treatment, chronic kidney disease, and/or type two diabetes [14, 46]. A recent review and meta-analysis including 15 trials showed that SGLT2i use is associated with fewer HF hospitalizations as well as a decreased cardiovascular mortality risk in patients with HF, type 2 diabetes, and chronic kidney failure [47]. Another meta-analysis focused on the effects of SGLT2i on albuminuria and found an approximate 25% decrease in UACR in normoalbuminuria, and up to 35–40% in patients with micro- or macroalbuminuria [48]. The responsible mechanism is believed to include anti-inflammatory effects, decreased production of reactive oxygen species, improvement of endothelial function, lowering blood pressure, promoting favorable cardiac remodeling, and lowering blood glucose in those with hyperglycaemia [49–53]. More specifically, SGLT2i lower the intraglomerular pressure through restoration of tubuloglomerular feedback, contributing directly to a decrease in albuminuria [54]. These synergistic effects explain the beneficial effect of SGLT2i across the entirety of the aforementioned patient population. The current study included merely a few patients using SGLT2i, thereby omitting analysis of its clinical effects in our study population. This is explained by the fact that most of the study timeframe took place prior to SGLT2i being incorporated into European guidelines on the treatment of HF.

### Study limitations

When interpreting the results of the current study, some limitations should be taken into account. Inherently to the study design, including patients who have been referred to our HFpEF outpatient clinic by another healthcare professional, selection bias cannot be excluded. The study population concerned ambulatory patients and ambulatory measurements, limiting generalizability to findings for patients with acute decompensated heart failure. Third, the current study consisted of relatively small groups, lacking sufficient statistical power to detect subtle differences between subgroups, e.g., prediabetes and macroalbuminuria. Similarly to medication use, small absolute numbers of prescriptions and in this case additional missing records omitted the ability to identify trends and an association with clinical outcomes.

## Conclusions

Impaired glucose metabolism and albuminuria are prevalent in HFpEF at baseline, and re-evaluation one year later still reveals new diagnoses. Both factors are associated with adverse outcomes. Albuminuria at any time point is associated with adverse outcomes, regardless of recovery from albuminuria one year later. It is to be elucidated in future studies whether treatments in patients with HFpEF reduce adverse outcomes through alteration of glucose metabolism or albuminuria, to optimize targeted therapies in HFpEF.

## Electronic supplementary material

Below is the link to the electronic supplementary material.

Supplementary Material 1

## Data Availability

Data is provided within the manuscript or supplementary information files. The datasets used and/or analysed during the current study are available from the corresponding author on reasonable request.

## Abbreviations

AF: Atrial fibrillation
ACEi: Angiotensin-converting enzyme inhibitor
aHR: Adjusted hazard ratio
ALB+: Albuminuria
ALB−: Normoalbuminuria
ANOVA: Analysis of variance
ARB: Angiotensin receptor blocker
BB: Beta blocker
BMI: Body mass index
CAD: Coronary artery disease
CCB: Calcium channel blocker
CI: Confidence interval
COPD: Chronic obstructive pulmonary disease
DM: Diabetes mellitus
DM+: Diabetes mellitus or prediabetes
DM−: Normoglycaemia
DMxALB: Diabetes mellitus with albuminuria interaction term
eGFR: Estimated glomerular filtration rate
GLP-1RA: Glucagon-like peptide-1 receptor agonists
HbA1c: Hemoglobin A1c
HR: Hazard ratio
IQR: Interquartile range
LAVI: Left atrial volume index
LVEF: Left ventricle ejection fraction
LVMI: Left ventricle mass index
MRA: Mineralcorticoid receptor antagonist
NT-proBNP: N-terminal prohormone brain natriuretic peptide
NYHA: New York Heart Association
RERI: Relative excess risk due to interaction
SGLT2i: Sodium–glucose co-transporter 2 inhibitor
SI: Synergy index
T0: Baseline visit at the outpatient clinic
T1: One year follow-up
UACR: Urinary albumin-creatinine ratio

## References

[1] Savarese G, Becher PM, Lund LH, Seferovic P, Rosano GMC, Coats AJS. Global burden of heart failure: a comprehensive and updated review of epidemiology. Cardiovasc Res. 2022;118:3272–87. 10.1093/cvr/cvac013 35150240

[2] Packer M, Lam CSP, Lund LH, Maurer MS, Borlaug BA. Characterization of the inflammatory-metabolic phenotype of heart failure with a preserved ejection fraction: a hypothesis to explain influence of sex on the evolution and potential treatment of the disease. Eur J Heart Fail. 2020;22:1551–67. 32441863 10.1002/ejhf.1902 PMC7687188

[3] Kristensen SL, Jhund PS, Lee MMY, Køber L, Solomon SD, Granger CB, Yusuf S, Pfeffer MA, Swedberg K, McMurray JJV. CHARM investigators and committees. Prevalence of prediabetes and undiagnosed diabetes in patients with HFpEF and HFrEF and associated clinical outcomes. Cardiovasc Drugs Ther. 2017;31:545–9. 28948430 10.1007/s10557-017-6754-x PMC5730631

[4] Johansson I, Dahlström U, Edner M, Näsman P, Rydén L, Norhammar A. Type 2 diabetes and heart failure: characteristics and prognosis in preserved, mid-range and reduced ventricular function. Diab Vasc Dis Res. 2018;15:494–503. 30176743 10.1177/1479164118794619

[5] Tribouilloy C, Rusinaru D, Mahjoub H, Tartière J-M, Kesri-Tartière L, Godard S, Peltier M. Prognostic impact of diabetes mellitus in patients with heart failure and preserved ejection fraction: a prospective five-year study. Heart Br Card Soc. 2008;94:1450–5. 10.1136/hrt.2007.128769 18208832

[6] Brannick B, Dagogo-Jack S. Prediabetes and cardiovascular disease: pathophysiology and interventions for prevention and risk reduction. Endocrinol Metab Clin North Am. 2018;47:33–50. 29407055 10.1016/j.ecl.2017.10.001 PMC5806140

[7] Ford ES, Zhao G, Li C. Pre-diabetes and the risk for cardiovascular disease: a systematic review of the evidence. J Am Coll Cardiol. 2010;55:1310–7. 20338491 10.1016/j.jacc.2009.10.060

[8] Sandesara PB, O’Neal WT, Kelli HM, Samman-Tahhan A, Hammadah M, Quyyumi AA, Sperling LS. The prognostic significance of diabetes and microvascular complications in patients with heart failure with preserved ejection fraction. Diabetes Care. 2018;41:150–5. 29051160 10.2337/dc17-0755 PMC5741155

[9] Khan MS, Shahid I, Anker SD, Fonarow GC, Fudim M, Hall ME, Hernandez A, Morris AA, Shafi T, Weir MR, Zannad F, Bakris GL, Butler J. Albuminuria and heart failure: JACC state-of-the-art review. J Am Coll Cardiol. 2023;81:270–82. 36653095 10.1016/j.jacc.2022.10.028

[10] Martens RJH, Houben AJHM, Kooman JP, Berendschot TTJM, Dagnelie PC, van der Kallen CJH, Kroon AA, Leunissen KML, van der Sande FM, Schaper NC, Schouten JSAG, Schram MT, Sep SJS, Sörensen BM, Henry RMA, Stehouwer CDA. Microvascular endothelial dysfunction is associated with albuminuria: the Maastricht study. J Hypertens. 2018;36:1178–87. 29373478 10.1097/HJH.0000000000001674

[11] Weerts J, Mourmans SG, Barandiarán Aizpurua A, Schroen BL, Knackstedt C, Eringa E, Houben AJ, van Empel VP. The role of systemic microvascular dysfunction in heart failure with preserved ejection fraction. Biomolecules. 2022;12:278. 35204779 10.3390/biom12020278 PMC8961612

[12] Volpe M, Gallo G. Cardiometabolic phenotype of heart failure with preserved ejection fraction as a target of sodium–glucose co-transporter 2 inhibitors and glucagon-like peptide receptor agonists. Cardiovasc Res. 2021;117:1992–4. 33231613 10.1093/cvr/cvaa334

[13] Bell DSH, Goncalves E. Heart failure in the patient with diabetes: epidemiology, aetiology, prognosis, therapy and the effect of glucose-lowering medications. Diabetes Obes Metab. 2019;21:1277–90. 30724013 10.1111/dom.13652

[14] Vaduganathan M, Docherty KF, Claggett BL, Jhund PS, de Boer RA, Hernandez AF, Inzucchi SE, Kosiborod MN, Lam CSP, Martinez F, Shah SJ, Desai AS, McMurray JJV, Solomon SD. SGLT-2 inhibitors in patients with heart failure: a comprehensive meta-analysis of five randomised controlled trials. Lancet Lond Engl. 2022;400:757–67. 10.1016/S0140-6736(22)01429-5 36041474

[15] Perkovic V, Tuttle KR, Rossing P, Mahaffey KW, Mann JFE, Bakris G, Baeres FMM, Idorn T, Bosch-Traberg H, Lausvig NL, Pratley R. FLOW trial committees and investigators. Effects of semaglutide on chronic kidney disease in patients with type 2 diabetes. N Engl J Med. 2024;391:109–21. 38785209 10.1056/NEJMoa2403347

[16] Patel R, Wadid M, Makwana B, Kumar A, Khadke S, Bhatti A, Banker A, Husami Z, Labib S, Venesy D, Fonarow G, Kosiborod M, Al-Kindi S, Bhatt DL, Dani S, Nohria A, Butler J, Ganatra S. GLP-1 receptor agonists among patients with overweight or obesity, diabetes, and HFpEF on SGLT2 inhibitors. JACC Heart Fail. 2024;12:1814–26. 39207323 10.1016/j.jchf.2024.07.006

[17] Barandiaran Aizpurua A, Sanders-van Wijk S, Brunner-La Rocca H-P, Henkens MT, Weerts J, Spanjers MH, Knackstedt C, van Empel VP. Iron deficiency impacts prognosis but less exercise capacity in heart failure with preserved ejection fraction. ESC Heart Fail. 2021;8:1304–13. 33522131 10.1002/ehf2.13204 PMC8006701

[18] Henkens MTHM, Weerts J, Verdonschot JAJ, Raafs AG, Stroeks S, Sikking MA, Amin H, Mourmans SGJ, Geraeds CBG, Sanders-van Wijk S, Barandiarán Aizpurua A, Uszko-Lencer NHMK, Krapels IPC, Wolffs PFG, Brunner HG, van Leeuwen REW, Verhesen W, Schalla SM, van Stipdonk AWM, Knackstedt C, Li X, Abdul Hamid MA, van Paassen P, Hazebroek MR, Vernooy K, Brunner-La Rocca H-P, van Empel VPM, Heymans SRB. Improving diagnosis and risk stratification across the ejection fraction spectrum: the Maastricht cardiomyopathy registry. ESC Heart Fail. 2022;9:1463–70. 35118823 10.1002/ehf2.13833 PMC8934928

[19] Ponikowski P, Voors AA, Anker SD, Bueno H, Cleland JGF, Coats AJS, Falk V, González-Juanatey JR, Harjola V-P, Jankowska EA, Jessup M, Linde C, Nihoyannopoulos P, Parissis JT, Pieske B, Riley JP, Rosano GMC, Ruilope LM, Ruschitzka F, Rutten FH, Meer P van der, ESC Scientific Document Group. ESC guidelines for the diagnosis and treatment of acute and chronic heart failure: the task force for the diagnosis and treatment of acute and chronic heart failure of the European society of cardiology (ESC)Developed with the special contribution of the heart failure association (HFA) of the ESC. Eur Heart J. 2016;2016(37):2129–200. 10.1093/eurheartj/ehw128 27206819

[20] McDonagh TA, Metra M, Adamo M, Gardner RS, Baumbach A, Böhm M, Burri H, Butler J, Čelutkienė J, Chioncel O, Cleland JGF, Coats AJS, Crespo-Leiro MG, Farmakis D, Gilard M, Heymans S, Hoes AW, Jaarsma T, Jankowska EA, Lainscak M, Lam CSP, Lyon AR, McMurray JJV, Mebazaa A, Mindham R, Muneretto C, Francesco Piepoli M, Price S, Rosano GMC, Ruschitzka F, Kathrine Skibelund A, ESC Scientific Document Group. ESC guidelines for the diagnosis and treatment of acute and chronic heart failure. Eur Heart J. 2021;2021(42):3599–726.

[21] Komajda M, Carson PE, Hetzel S, McKelvie R, McMurray J, Ptaszynska A, Zile MR, Demets D, Massie BM. Factors associated with outcome in heart failure with preserved ejection fraction: findings from the Irbesartan in heart failure with preserved ejection fraction study (I-PRESERVE). Circ Heart Fail. 2011;4:27–35. 21068341 10.1161/CIRCHEARTFAILURE.109.932996

[22] Weerts J, Barandiarán Aizpurua A, Henkens MT, Lyon A, van Mourik MJ, van Gemert MR, Raafs A, Sanders-van Wijk S, Bayés-Genís A, Heymans SR. The prognostic impact of mechanical atrial dysfunction and atrial fibrillation in heart failure with preserved ejection fraction. Eur Heart J-Cardiovasc Imaging. 2022;23:74–84. 10.1093/ehjci/jeab222 PMC8685598 34718457

[23] Harrell FE. Regression modeling strategies: with applications to linear models, logistic and ordinal regression, and survival analysis. Cham: Springer International Publishing; 2015.

[24] Zheng R, Qian S, Shi Y, Lou C, Xu H, Pan J. Association between triglyceride-glucose index and in-hospital mortality in critically ill patients with sepsis: analysis of the MIMIC-IV database. Cardiovasc Diabetol. 2023;22:307. 37940931 10.1186/s12933-023-02041-w PMC10634031

[25] Selvaraj S, Claggett B, Shah SJ, Anand I, Rouleau JL, O’Meara E, Desai AS, Lewis EF, Pitt B, Sweitzer NK, Fang JC, Pfeffer MA, Solomon SD. Prognostic value of albuminuria and influence of spironolactone in heart failure with preserved ejection fraction. Circ Heart Fail. 2018;11: e005288. 30571191 10.1161/CIRCHEARTFAILURE.118.005288 PMC6594383

[26] Katz DH, Burns JA, Aguilar FG, Beussink L, Shah SJ. Albuminuria is independently associated with cardiac remodeling, abnormal right and left ventricular function, and worse outcomes in heart failure with preserved ejection fraction. JACC Heart Fail. 2014;2:586–96. 25282032 10.1016/j.jchf.2014.05.016 PMC4256131

[27] Mgbemena O, Zhang Y, Velarde G. Role of diabetes mellitus in heart failure with preserved ejection fraction: a review article. Cureus. 2021;13: e19398. 34926000 10.7759/cureus.19398 PMC8654084

[28] Phang RJ, Ritchie RH, Hausenloy DJ, Lees JG, Lim SY. Cellular interplay between cardiomyocytes and non-myocytes in diabetic cardiomyopathy. Cardiovasc Res. 2023;119:668–90. 35388880 10.1093/cvr/cvac049 PMC10153440

[29] McHugh K, DeVore AD, Wu J, Matsouaka RA, Fonarow GC, Heidenreich PA, Yancy CW, Green JB, Altman N, Hernandez AF. Heart failure with preserved ejection fraction and diabetes: JACC state-of-the-art review. J Am Coll Cardiol. 2019;73:602–11. 30732715 10.1016/j.jacc.2018.11.033

[30] Son TK, Toan NH, Thang N, Le Trong TH, Tien HA, Thuy NH, Van Minh H, Valensi P. Prediabetes and insulin resistance in a population of patients with heart failure and reduced or preserved ejection fraction but without diabetes, overweight or hypertension. Cardiovasc Diabetol. 2022;21:75. 35568879 10.1186/s12933-022-01509-5 PMC9107647

[31] Jackson AM, Rørth R, Liu J, Kristensen SL, Anand IS, Claggett BL, Cleland JGF, Chopra VK, Desai AS, Ge J, Gong J, Lam CSP, Lefkowitz MP, Maggioni AP, Martinez F, Packer M, Pfeffer MA, Pieske B, Redfield MM, Rizkala AR, Rouleau JL, Seferović PM, Tromp J, Van Veldhuisen DJ, Yilmaz MB, Zannad F, Zile MR, Køber L, Petrie MC, Jhund PS, Solomon SD, McMurray JJV. PARAGON-HF committees and investigators. Diabetes and pre-diabetes in patients with heart failure and preserved ejection fraction. Eur J Heart Fail. 2022;24:497–509. 34918855 10.1002/ejhf.2403 PMC9542636

[32] Gerstein HC, Mann JF, Yi Q, Zinman B, Dinneen SF, Hoogwerf B, Hallé JP, Young J, Rashkow A, Joyce C, Nawaz S, Yusuf S. HOPE study investigators. Albuminuria and risk of cardiovascular events, death, and heart failure in diabetic and nondiabetic individuals. JAMA. 2001;286:421–6. 11466120 10.1001/jama.286.4.421

[33] Yamanouchi M, Sawa N, Toyama T, Shimizu M, Oshima M, Yoshimura Y, Sugimoto H, Kurihara S, Oba Y, Ikuma D, Mizuno H, Sekine A, Suwabe T, Hoshino J, Ubara Y, Hara S, Furuichi K, Wada T. Trajectory of GFR decline and fluctuation in albuminuria leading to end-stage kidney disease in patients with biopsy-confirmed diabetic kidney disease. Kidney Int Rep. 2024;9:323–33. 38344735 10.1016/j.ekir.2023.11.004 PMC10851062

[34] Weir MR. Microalbuminuria and cardiovascular disease. Clin J Am Soc Nephrol CJASN. 2007;2:581–90. 17699466 10.2215/CJN.03190906

[35] Paulus WJ, Tschöpe C. A novel paradigm for heart failure with preserved ejection fraction: comorbidities drive myocardial dysfunction and remodeling through coronary microvascular endothelial inflammation. J Am Coll Cardiol. 2013;62:263–71. 23684677 10.1016/j.jacc.2013.02.092

[36] de Jong PE, Hillege HL, Pinto-Sietsma SJ, de Zeeuw D. Screening for microalbuminuria in the general population: a tool to detect subjects at risk for progressive renal failure in an early phase? Nephrol Dial Transpl Off Publ Eur Dial Transpl Assoc Eur Ren Assoc. 2003;18:10–3. 10.1093/ndt/18.1.10 12480951

[37] Shin J-I, Chang AR, Grams ME, Coresh J, Ballew SH, Surapaneni A, Matsushita K, Bilo HJG, Carrero JJ, Chodick G, Daratha KB, Jassal SK, Nadkarni GN, Nelson RG, Nowak C, Stempniewicz N, Sumida K, Traynor JP, Woodward M, Sang Y, Gansevoort RT. CKD prognosis consortium albuminuria testing in hypertension and diabetes: an individual-participant data meta-analysis in a global consortium. Hypertens Dallas Tex. 1979;2021(78):1042–52. 10.1161/HYPERTENSIONAHA.121.17323 PMC8429211 34365812

[38] Boorsma EM, Ter Maaten JM, Damman K, van Essen BJ, Zannad F, van Veldhuisen DJ, Samani NJ, Dickstein K, Metra M, Filippatos G, Lang CC, Ng L, Anker SD, Cleland JG, Pellicori P, Gansevoort RT, Heerspink HJL, Voors AA, Emmens JE. Albuminuria as a marker of systemic congestion in patients with heart failure. Eur Heart J. 2023;44:368–80. 36148485 10.1093/eurheartj/ehac528 PMC9890244

[39] Jackson CE, Solomon SD, Gerstein HC, Zetterstrand S, Olofsson B, Michelson EL, Granger CB, Swedberg K, Pfeffer MA, Yusuf S, McMurray JJV. CHARM investigators and committees. Albuminuria in chronic heart failure: prevalence and prognostic importance. Lancet Lond Engl. 2009;374:543–50. 10.1016/S0140-6736(09)61378-7 19683640

[40] Solomon SD, McMurray JJV, Vaduganathan M, Claggett B, Jhund PS, Desai AS, Henderson AD, Lam CSP, Pitt B, Senni M, Shah SJ, Voors AA, Zannad F, Abidin IZ, Alcocer-Gamba MA, Atherton JJ, Bauersachs J, Chang-Sheng M, Chiang C-E, Chioncel O, Chopra V, Comin-Colet J, Filippatos G, Fonseca C, Gajos G, Goland S, Goncalvesova E, Kang S, Katova T, Kosiborod MN, Latkovskis G. Finerenone in heart failure with mildly reduced or preserved ejection fraction. N Engl J Med. 2024;391:1475–85.39225278

[41] Mc Causland FR, Vaduganathan M, Claggett BL, Kulac IJ, Desai AS, Jhund PS, Henderson AD, Brinker M, Perkins R, Scheerer MF, Schloemer P, Lam CSP, Senni M, Shah SJ, Voors AA, Zannad F, Pitt B, McMurray JJV, Solomon SD. Finerenone and kidney outcomes in patients with heart failure: the FINEARTS-HF trial. J Am Coll Cardiol. 2025;85:159–68. 39490700 10.1016/j.jacc.2024.10.091

[42] Levey AS, Eckardt K-U, Dorman NM, Christiansen SL, Cheung M, Jadoul M, Winkelmayer WC. Nomenclature for kidney function and disease-executive summary and glossary from a kidney disease: improving global outcomes (KDIGO) consensus conference. Eur Heart J. 2020;41:4592–8. 33141221 10.1093/eurheartj/ehaa650 PMC7774468

[43] Blecker S, Matsushita K, Köttgen A, Loehr LR, Bertoni AG, Boulware LE, Coresh J. High-Normal albuminuria and risk of heart failure in the community. Am J Kidney Dis Off J Natl Kidney Found. 2011;58:47–55. 10.1053/j.ajkd.2011.02.391 PMC3119712 21549463

[44] Pitt B, Pfeffer MA, Assmann SF, Boineau R, Anand IS, Claggett B, Clausell N, Desai AS, Diaz R, Fleg JL, Gordeev I, Harty B, Heitner JF, Kenwood CT, Lewis EF, O’Meara E, Probstfield JL, Shaburishvili T, Shah SJ, Solomon SD, Sweitzer NK, Yang S, McKinlay SM. Spironolactone for heart failure with preserved ejection fraction. N Engl J Med. 2014;370:1383–92. 24716680 10.1056/NEJMoa1313731

[45] Trujillo H, Caravaca-Fontán F, Caro J, Morales E, Praga M. The forgotten antiproteinuric properties of diuretics. Am J Nephrol. 2021;52:435–49. 34233330 10.1159/000517020

[46] McDonagh TA, Metra M, Adamo M, Gardner RS, Baumbach A, Böhm M, Burri H, Butler J, Čelutkienė J, Chioncel O, Cleland JGF, Crespo-Leiro MG, Farmakis D, Gilard M, Heymans S, Hoes AW, Jaarsma T, Jankowska EA, Lainscak M, Lam CSP, Lyon AR, McMurray JJV, Mebazaa A, Mindham R, Muneretto C, Francesco Piepoli M, Price S, Rosano GMC, Ruschitzka F, Skibelund AK, ESC Scientific Document Group. 2023 Focused Update of the 2021 ESC Guidelines for the diagnosis and treatment of acute and chronic heart failure: Developed by the task force for the diagnosis and treatment of acute and chronic heart failure of the European Society of Cardiology (ESC) With the special contribution of the Heart Failure Association (HFA) of the ESC. *Eur Heart J* 2023:ehad195. 10.1093/eurheartj/ehab670 34649282

[47] Usman MS, Bhatt DL, Hameed I, Anker SD, Cheng AYY, Hernandez AF, Jones WS, Khan MS, Petrie MC, Udell JA, Friede T, Butler J. Effect of SGLT2 inhibitors on heart failure outcomes and cardiovascular death across the cardiometabolic disease spectrum: a systematic review and meta-analysis. Lancet Diabetes Endocrinol. 2024;12:447–61. 38768620 10.1016/S2213-8587(24)00102-5

[48] Piperidou A, Sarafidis P, Boutou A, Thomopoulos C, Loutradis C, Alexandrou ME, Tsapas A, Karagiannis A. The effect of SGLT-2 inhibitors on albuminuria and proteinuria in diabetes mellitus: a systematic review and meta-analysis of randomized controlled trials. J Hypertens. 2019;37:1334–43. 31145707 10.1097/HJH.0000000000002050

[49] Uthman L, Baartscheer A, Bleijlevens B, Schumacher CA, Fiolet JWT, Koeman A, Jancev M, Hollmann MW, Weber NC, Coronel R, Zuurbier CJ. Class effects of SGLT2 inhibitors in mouse cardiomyocytes and hearts: inhibition of Na+/H+ exchanger, lowering of cytosolic Na+ and vasodilation. Diabetologia. 2018;61:722–6. 29197997 10.1007/s00125-017-4509-7 PMC6448958

[50] Juni RP, Al-Shama R, Kuster DWD, Velden J, Hamer HM, Vervloet MG, Eringa EC, Koolwijk P, Hinsbergh VWM. Empagliflozin restores chronic kidney disease–induced impairment of endothelial regulation of cardiomyocyte relaxation and contraction. Kidney Int. 2021;99:1088–101. 33359500 10.1016/j.kint.2020.12.013

[51] Benetti E, Mastrocola R, Vitarelli G, Cutrin JC, Nigro D, Chiazza F, Mayoux E, Collino M, Fantozzi R. Empagliflozin protects against diet-induced NLRP-3 inflammasome activation and lipid accumulation. J Pharmacol Exp Ther. 2016;359:45–53. 27440421 10.1124/jpet.116.235069

[52] Verma S, McMurray JJV. SGLT2 inhibitors and mechanisms of cardiovascular benefit: a state-of-the-art review. Diabetologia. 2018;61:2108–17. 30132036 10.1007/s00125-018-4670-7

[53] Chilton R, Tikkanen I, Cannon CP, Crowe S, Woerle HJ, Broedl UC, Johansen OE. Effects of empagliflozin on blood pressure and markers of arterial stiffness and vascular resistance in patients with type 2 diabetes. Diabetes Obes Metab. 2015;17:1180–93. 26343814 10.1111/dom.12572 PMC5057299

[54] Sen T, Heerspink HJL. A kidney perspective on the mechanism of action of sodium glucose co-transporter 2 inhibitors. Cell Metab. 2021;33:732–9. 33691091 10.1016/j.cmet.2021.02.016

