# Supplementary material for: Temporal prevalence and prognostic impact of diabetes mellitus and albuminuria in heart failure with preserved ejection fraction
Source: Cardiovasc Diabetol. 2025 Apr 5;24:156. doi: 10.1186/s12933-025-02708-6 (PMC11972526; doi:10.1186/s12933-025-02708-6)
Supplement: Supplementary file 1 — Supplementary Material 1 [file 12933_2025_2708_MOESM1_ESM.docx]

**Electronic supplementary material**

**Supplemental methods**

For multiplicative interaction, we added an interaction term (DMxALB) to the Cox model and assessed its significance. Additive interaction was analyzed using three metrics: the relative excess risk due to interaction (RERI), which indicates whether the combined effect exceeds the sum of individual effects; the synergy index (SI), where SI>1 denotes synergy and SI<1 indicates antagonism; and the attributable proportion, which estimates the proportion of excess risk attributable to the combined exposure.

**Supplemental results**

Results from the multiplicative interaction analysis showed that the interaction term (ALBxDM) was positive though not statistically significant, with HR 1.04 (95% CI 0.44-2.48) and aHR 0.93 (95% CI 0.39-2.25). The additive interaction analysis indicated a positive but non-significant interaction effect, with a RERI of 0.82 (95% CI -0.89-2.53), suggesting a small excess risk due to the interaction; approximately 23% (attributable proportion 0.23 (95% CI -0.25-0.72). The synergy index was 1.48 (95% CI 0.31-2.66), suggesting a slight positive interaction.

**Supplemental tables**

**Table 1** Prevalence of HFH and mortality across subgroups at three years follow-up

|  | ALB- to ALB- N=128 | ALB- to ALB+ N=36 | ALB+ to ALB- N=27 | ALB+ to ALB+ N=59 | *p*-value |
| --- | --- | --- | --- | --- | --- |
| HFH or mortality | 11 (8.6%) | 5 (13.9%) | 6 (22.2%) | 16 (27.1%) | 0.008 |
| HFH | 7 (5.5%) | 2 (5.6%) | 4 (14.8%) | 11 (18.6%) | 0.022 |
| Mortality | 6 (4.7%) | 4 (11.1%) | 4 (14.8%) | 11 (18.6%) | 0.022 |

ALB-: normoalbuminuria, ALB+: albuminuria, HFH: heart failure hospitalization.

**Table 2** Medication use before the first outpatient clinic visit (baseline) and after one year follow-up in patients whom transitioned from albuminuria to normoalbuminuria (n=27)

|  | Baseline | One year FUP |
| --- | --- | --- |
| ACEi/ARB n(%) | 16 (59.3) | 7 (46.7) |
| MRA n(%) | 5 (18.5) | 4 (26.7) |
| SGLT2i n(%) | 0 (0.0) | 1 (6.7) |
| BB n(%) | 17 (63.0) | 5 (35.7) |
| Loop diuretic n(%) | 21 (77.8) | 11 (73.3) |
| CCB n(%) | 17 (63.0) | 6 (40.0) |

ACEi: angiotensin-converting enzyme inhibitor, ARB: angiotensin receptor blocker, BB: beta blocker, CCB: calcium channel blocker, FUP: follow-up, MRA: mineralcorticoid receptor antagonist, SGLT2i: sodium-glucose co-transporter 2 inhibitor.

**Supplementary figures
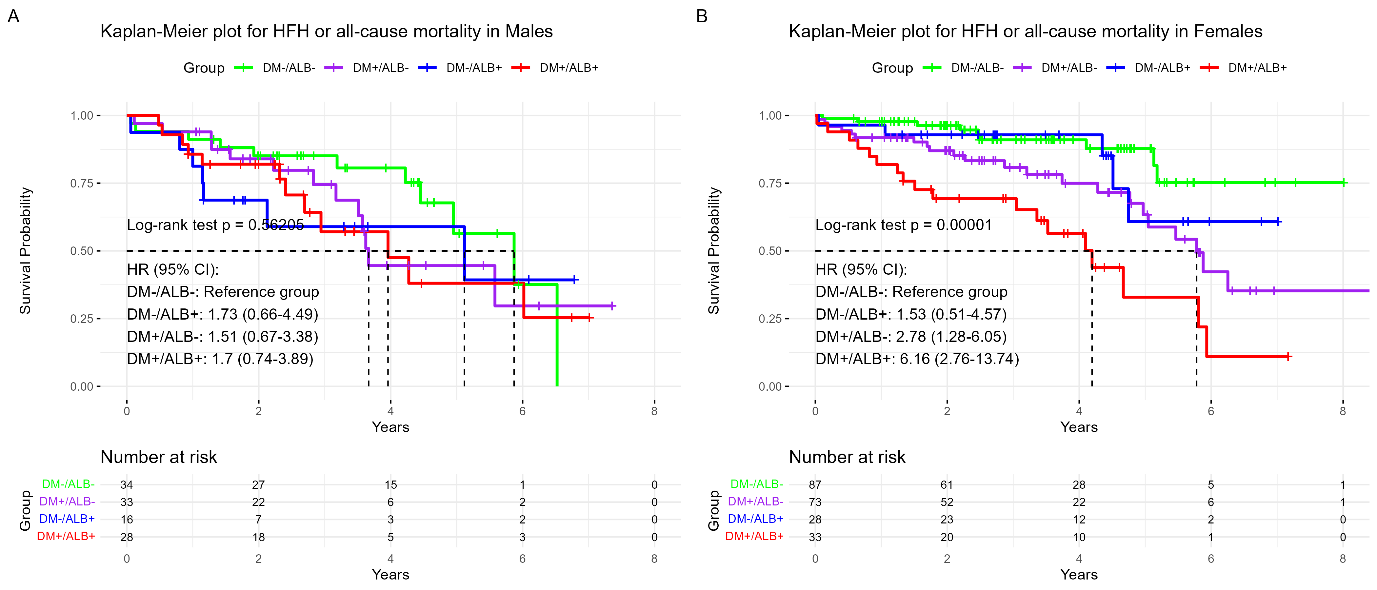
**

**Fig. 1** Sex-specific Kaplan-Meier plots.
A: KM plot in males. B: KM plot in females.
Dotted lines represent 50% of patients with event-free survival. The Schoenfeld residuals tests yielded χ² = 3.59 (df=3, *p*=0.31) for males, and χ² = 2.4 (df=3, *p*=0.49) for females, indicating proportional hazards assumption is satisfied globally for the model and assumptions of the log-rank test are met.
ALB: albuminuria, DM: diabetes mellitus, KM: Kaplan-Meier


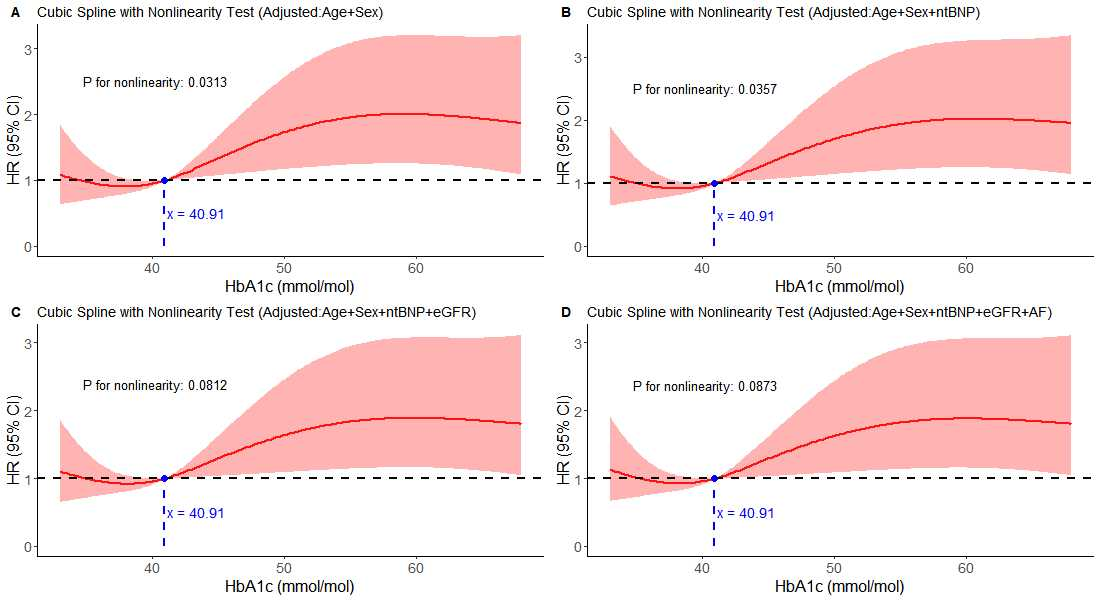
**Fig. 2** Adjusted Restricted cubic spline plot of HbA1c
AF: atrial fibrillation, eGFR: estimated glomerular filtration rate, HbA1c: hemoglobin A1c, HR: hazard ratio, NT-proBNP: N-terminal prohormone brain natriuretic peptide, UACR: urinary albumin-creatinine ratio.


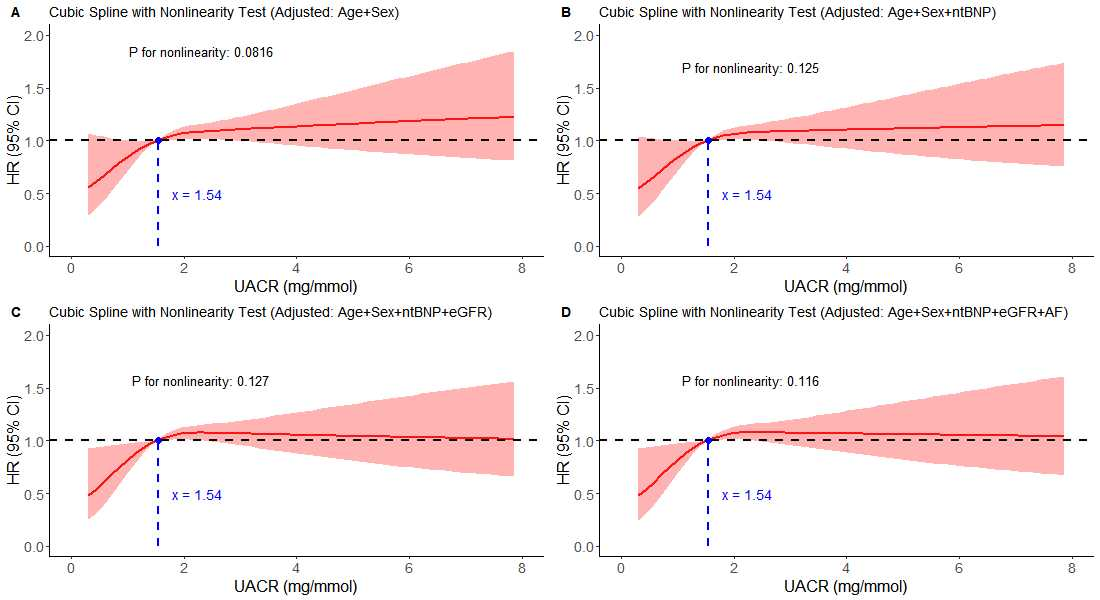
**Fig. 3** Adjusted Restricted cubic spline plot of UACR.
AF: atrial fibrillation, eGFR: estimated glomerular filtration rate, HbA1c: hemoglobin A1c, HR: hazard ratio, NT-proBNP: N-terminal prohormone brain natriuretic peptide, UACR: urinary albumin-creatinine ratio.
